# Evolution and clinical significance of HER2-low status after neoadjuvant therapy for breast cancer

**DOI:** 10.3389/fonc.2023.1086480

**Published:** 2023-02-22

**Authors:** Jiuyan Shang, Xuemei Sun, Zihang Xu, Lijing Cai, Chang Liu, Si Wu, Yueping Liu

**Affiliations:** ^1^ Department of Pathology, Hebei Medical University, The Fourth Affiliated Hospital and Hebei Provincial Tumor Hospital, Shijiazhuang, China; ^2^ Anesthesia Class, School of Basic Medicine, Hebei Medical University, Shijiazhuang, China

**Keywords:** breast cancer, human epidermal growth factor receptor 2, hormone receptor, neoadjuvant therapy, pathological complete response

## Abstract

**Background:**

The emergence of HER2 antibody-drug conjugates provides new treatment decisions for breast cancer patients, especially those with HER2-low expression. In order to explore the biological characteristics of breast cancer with HER2-low expression, the HER2-low category in primary breast cancer and residual tumor after neoadjuvant therapy was investigated to reflect the evolution of HER2 expression.

**Methods:**

HER2 was assessed according to the latest ASCO/CAP guidelines. The cut-off value for staining of HER2-positive cells was >10%. HER2-negative cases were divided into HER2-low (IHC=1+/2+ and no ISH amplification) and HER2-zero (IHC-0), and the clinicopathological characteristics of the cases were collected.

**Results:**

This study included 1140 patients with invasive breast cancer who received preoperative neoadjuvant therapy from 2018 to 2021, of which 365 patients achieved pCR and 775 were non-pCR. In the non-pCR cohort, HER2-low cases accounted for 59.61% of primary tumors and 55.36% of residual tumors. Among HER2-negative cases, HR-positive tumors had a higher incidence of low HER2 expression compared with triple-negative tumors (80.27% vs 60.00% in primary tumors and 72.68% vs 50.77% in residual tumors). The inconsistency rate of HER2 expression was 21.42%, mainly manifested as the conversion of HER2-low cases to HER2-zero (10.19%) and the conversion of HER2-zero to HER2-low (6.45%). Among the HER2-negative cases in the primary tumor, the HER2 discordance rate of HR-positive cases was lower than that of triple-negative cases (23.34% VS 36.92%). This difference was mainly caused by the case switching from HER2-low to HER2-zero. Compared with HER2-zero cases, there were statistically significant differences in RCB grade, MP grade and the number of metastatic lymph nodes in HER2-low cases. Patients with low HER2 expression had a lower pathological response rate and a higher number of metastatic lymph nodes.

**Conclusion:**

HER2-low breast cancer is highly unstable during disease evolution and has certain biological characteristics. HER2-low breast cancer is not only correlated with positive HR, but also has a certain correlation with positive AR. Re-detection of HER2 in breast cancer after neoadjuvant therapy may lead to new treatment opportunities for a certain proportion of patients.

## Introduction

Human epidermal growth factor receptor-2 (HER2) is a proto-oncogene and has a high response rate in breast cancer and other types of cancers. Beyond that, HER2 status defines a distinct breast cancer subtype with aggressive biological behavior and historically worse prognosis, a reality that was changed after the incorporation of HER2 therapy (1). 2018 American Society of Clinical Oncology/College of American Pathologists (ASCO/CAP) guidelines recommend a binary distinction between HER2-positive and HER2-negative breast cancers to guide clinicians’ treatment decision. However, the emergence of the antibody-drug conjugates (ADCs) has provided new treatment decisions for patients with low HER2 expression. Breast cancer classified as negative in a certain proportion (approximately 45-55%) (2–4) actually belong to the newly proposed HER2-low. Breast cancer with an immunohistochemistry (IHC) score of 1+ or 2+ and unamplified by *in situ* hybridization (ISH) is referred to as HER2-low breast cancer. Recently, the results of non-randomized trials with novel antibody–drug conjugates targeting HER2 (trastuzumab–deruxtecan and trastuzumab–duocarmazine) have suggested a level of efficacy in HER2-low patients with advanced breast cancer, with objective response rates ranging between 32% and 37% in a heavily pretreated population (5, 6). Trastuzumab deruxtecan (DS8201a), for instance, has achieved an objective response rate (ORR) of 37% in highly pretreated patients with HER2-low metastatic breast cancer (5), whereas in a similar population ORR with trastuzumab duocarmazine (SYD985) was 28-40% depending on HR expression (6). This led to the hypothesis that HER2-low tumors might represent a separate disease subset, distinct from other luminal and triple-negative breast cancers (TNBC). Indeed, several trials are currently exploring the potential of anti-HER2 agents in HER2-low patients.

In this study, the evolution and clinicopathological characteristics of HER2-low expression tumors were analyzed based on neoadjuvant breast cancer patients in China.

## Materials and methods

### General information

A total of 1140 patients who received preoperative neoadjuvant therapy in the Fourth Hospital of Hebei Medical University from January 2018 to December 2021 were screened, and all patients underwent surgery in this hospital. Neoadjuvant therapy includes preoperative chemotherapy and endocrine therapy. Of these, 775 patients did not achieve pathological complete response (pCR), and 365 patients achieved pCR. The clinicopathological characteristics of the cases were collected and analyzed. In the non-pCR cohort, there were 773 females and 2 males, ranging in age from 24 to 86 years old. In the pCR cohort, there were 365 females and 0 males. Two or more attending pathologists performed double-blind follow-up on hematoxylin-eosin (HE) sections and HER2 IHC sections of all patients to improve the clinicopathological data.

### Methods

Retrospective analysis of patients with breast cancer that met the criteria was performed by IHC and ISH. The IHC method used Roche’s rabbit monoclonal primary antibody and the BenchMarK XT automatic IHC instrument was used for detection. The clinicopathological data of the non-pCR cohort of patients were collected, and the biological characteristics of HER2 low expression cases and HER2 0 cases were analyzed.

### Interpretation criteria

According to the ASCO/CAP guidelines (7), HER2 IHC staining results were determined, HER2 0: no staining is observed HER2-null or membrane staining that is incomplete and is faint/barely perceptible and in <10% tumor cells; HER2 1+: incomplete membrane staining that is faint/barely perceptible and in >10% of tumor cells; HER2 2+: weak to moderate complete membrane staining in >10% of tumor cells; or circumferential membrane staining that is complete, intense, and in ≤10% of tumor cells; HER2 3+: circumferential membrane staining that is complete, intense, and in >10% of tumor cells. For HER2 2+ cases, the ISH method was used for further testing, where HER2-zero was determined as HER2 negative; 1+ and 2+ with no ISH amplification as HER2-low, 2+ with ISH amplification and 3+ as HER2 positive. Hormone receptor (HR)-positive, at least 1% of infiltrating tumor cells showed immunostaining. Androgen receptor (AR)-positive, at least 1% of infiltrating tumor cells showed immunostaining.

### Statistical methods

Statistical software SPSS 23.00 was used for statistical analysis and processing, Kappa was used for consistency analysis, and χ2 test was used to test the significance of differences. P<0.05 was considered statistically significant.

## Results

### Clinical data

1140 breast cancer patients after neoadjuvant therapy were collected, including 1138 females and 2 males, aged 24-86 years. There were 775 patients with invasive breast cancer in the non-pCR group, including 405 left breast masses, 368 right breast masses, and 2 double breast masses. Among the HER2-negative cases, 505 were invasive ductal carcinoma after neoadjuvant therapy, 7 were mucinous adenocarcinoma, and 62 were of undetermined type; 583 were <70 years old, and 10 were ≥70 years old. Residual cancer burden (RCB) Grading: 63 cases of grade I, 172 cases of grade II, and 358 cases of grade III; Miller Payen classification (MP classification): grades 1-5 were 9, 71, 425, 53, and 12 cases, respectively.

### Consistent analysis of HER2 status after neoadjuvant therapy

**Figure 1:**
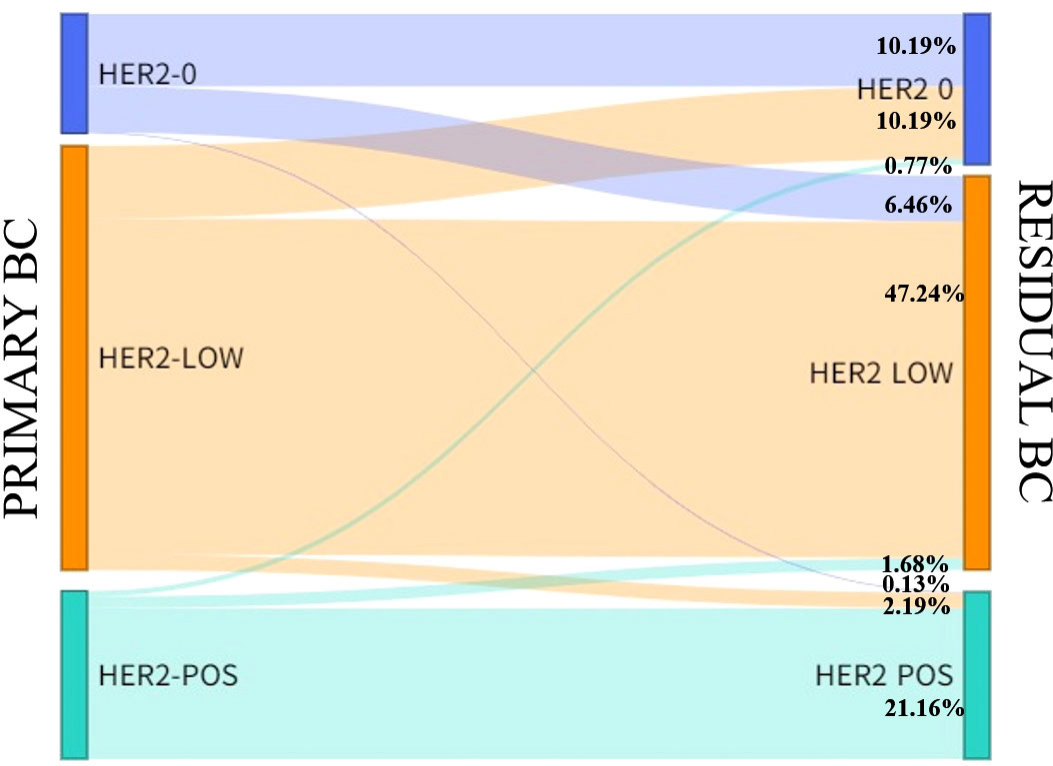
HER2 expression evolution from primary breast cancer to residual breast cancer.

**Figure 2:**
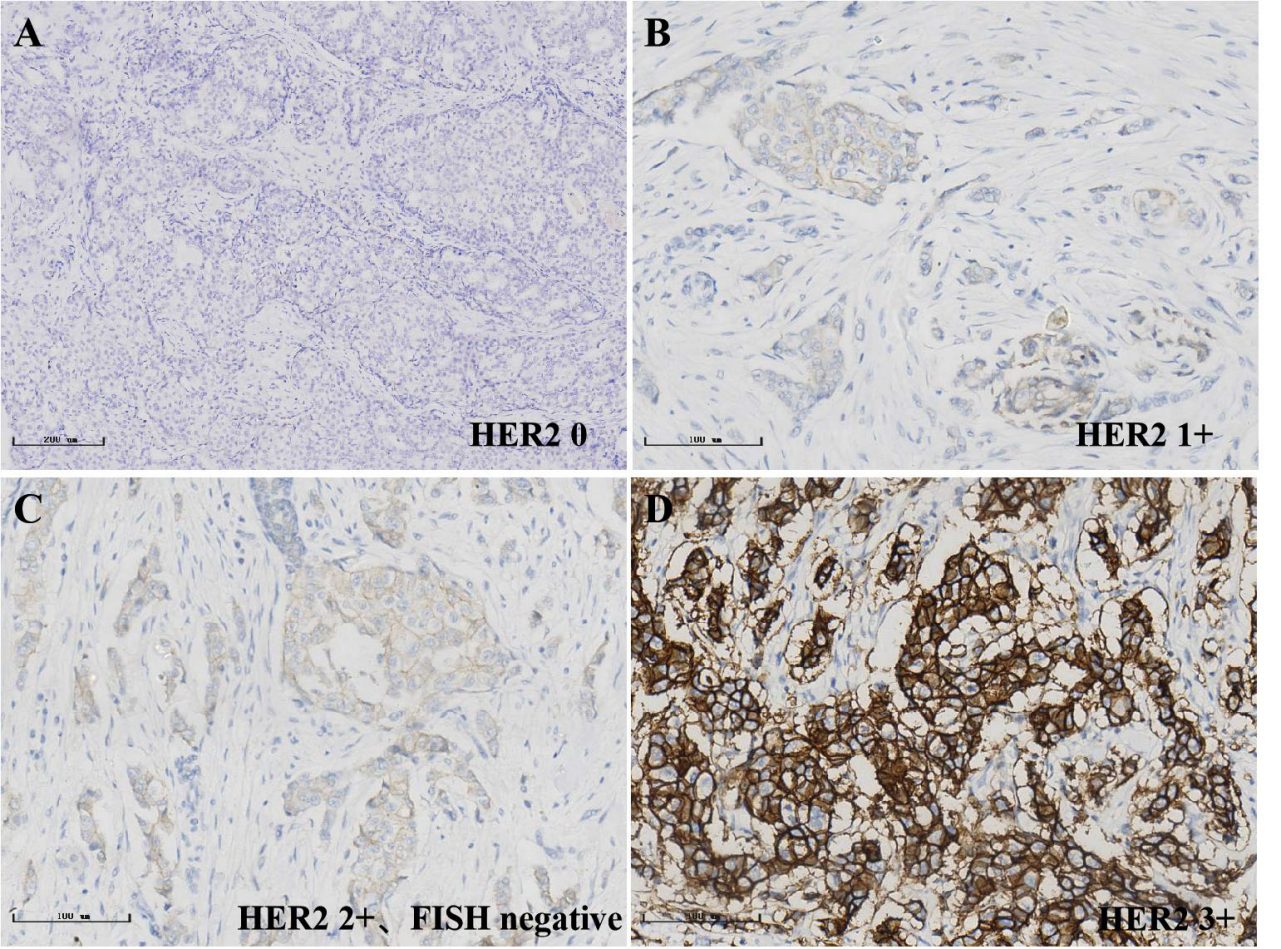
Immunohistochemical detection of HER2 status in breast cancer, streptavidin-perosidase (SP); **(A)**0; **(B)**1+; **(C)**2+; **(D)**3+.

**Figure 3:**
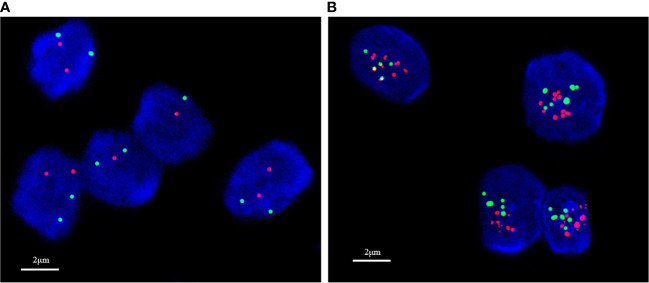
*In situ* hybridization detection of HER2 status in breast cancer. **(A)** No amplification; **(B)** amplification.

775 patients with invasive breast cancer were all tested for HER2. The interpretation was based on the ASCO guidelines. HER2-negative cases were divided into HER2-low (IHC=1+/2+ and no ISH amplification) and HER2-zero (IHC- 0). HER2 status of primary tumors: 130 cases of HER2-zero, 462 cases of HER2-low, and 183 cases of HER2-positive; HER2 status of residual tumors after neoadjuvant therapy: 164 cases of HER2-zero, 429 cases of HER2-low, and 182 cases of HER2-positive (**Table 1**). There was indeed a difference in the HER2 status of breast cancer before and after neoadjuvant therapy, and the difference was statistically significant (P=0.014), and the HER2 status was inconsistent (Kappa=0.630, P<0.001). The inconsistency rate was 21.42%, and the main difference: cases of HER2-low were switched to HER2-zero (**Figures 1**–**3**).

**Table 1:** HER2 expression evolution from primary breast cancer to residual breast cancer.

	HER2 expression on residual breast cancer n (%)			
	HER2-zero	HER2-low	HER2-pos	Total
HER2 expression on primary breast cancer n(%)				
HER2-zero	79 (10.19)	50 (6.46)	1 (0.13)	130 (16.78)
HER2-low	79 (10.19)	366 (47.23)	17 (2.19)	462 (59.61)
HER2-pos	6 (0.77)	13 (1.68)	164 (21.16)	183 (23.61)
Total	164 (21.16)	429 (55.36)	182 (23.48)	775

### HER2 low expression status and HR status

**Figure 4:**
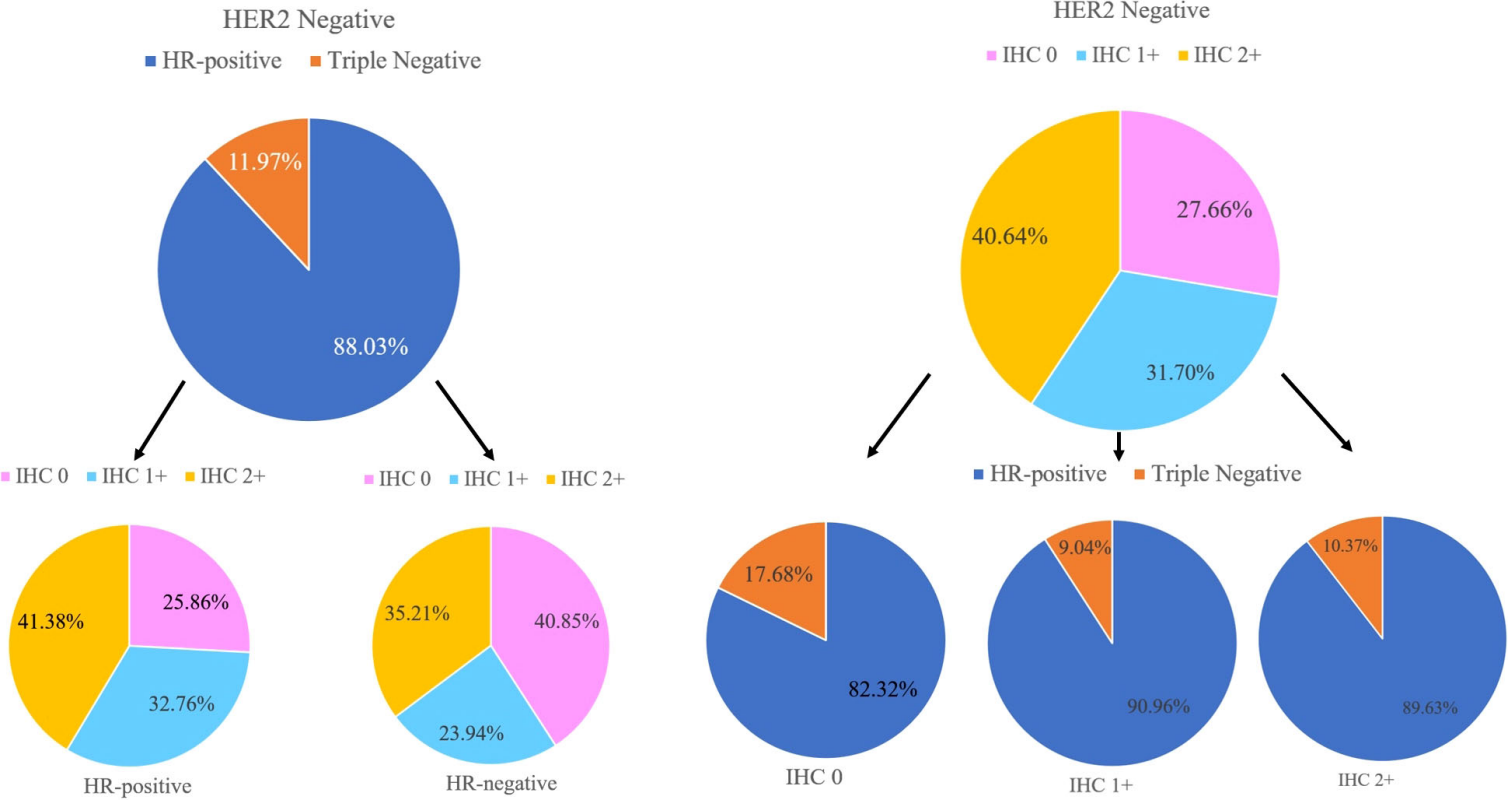
Hormone receptor (HR) status, HER2-low status, and IHC scores distributions within the HER2-negative population.

In the non-pCR cohort (N=775), HER2-low cases accounted for 59.61% (n=462) of primary breast cancer, 55.36% (n=429) of residual breast cancer after neoadjuvant therapy, respectively 78.04% and 71.92% of HER2-negative primary and residual breast cancers. In the analysis of HER2-negative cases, 527 were HR-positive cases and 65 were HR-negative cases among the primary breast cancers. Among the residual breast cancers after neoadjuvant therapy, there were 512 HR-positive cases and 62 HR-negative cases. The low expression of HER2 was 71.45% and 6.59% in the HR-positive/HER2-negative cohort and triple-negative cohort of primary breast cancer, respectively (p<0.01), and the residual breast cancer HR-positive/HER2-negative cohort and triple-negative cohort after neoadjuvant therapy were 66.72% and 5.75% respectively (p <0.01). After statistical chi-square test, low HER2 expression was positively correlated with HR-positive/HER2-negative breast cancer subtypes, and the difference was statistically significant (p<0.05) (**Table 2**). Compared with TNBC, the incidence of HER2-low tumors was higher in HR-positive tumors (80.27% vs. 60.00%; p<0.01). HR-positive tumors were characterized by a higher incidence of IHC 1+ and 2+ than TNBC (32.76% vs. 23.94% and 41.38% vs. 35.21%; p<0.05) (**Figure 4**).

**Table 2:** HER2 expression distribution according to breast cancer subtype in the HER2-negative primary and residual breast cancer cohort.

	HER2 expression n (%)		
	0	Low	*p*
Primary breast cancer n (%)			
HR-positive/HER2-negative	104 (17.57)	423 (71.45)	0.000*
Triple-negative	26 (4.39)	39 (6.59)	
Total	130	462	
Residual breast cancer n (%)			
HR-positive/HER2-negative	129 (22.47)	383 (66.72)	0.000*
Triple-negative	29 (5.05)	33 (5.75)	
Total	158	416	

*, P<0.01.

Analysis of HER2 1+ and HER2 2+ and HR status after neoadjuvant therapy in HER2-low cases, after statistical analysis, there was no statistical difference in HR status between HER2 1+ and HER2 2+ (P>0.05) (**Table 3**).

**Table 3:** Distribution of HER2 expression by IHC according to tumor phenotype in the HER2-low cohort.

	HER2 expression n (%)		
	1+	2+	*p*
Primary breast cancer n (%)			
HR-positive/HER2-negative	141 (30.52)	282 (61.04)	0.435
Triple-negative	14 (3.03)	25 (5.41)	
Total	155	307	
Residual breast cancer n (%)			
HR-positive/HER2-negative	136 (32.69)	247 (59.38)	0.067
Triple-negative	17 (4.09)	16 (3.85)	
Total	153	263	

**Figure 5:**
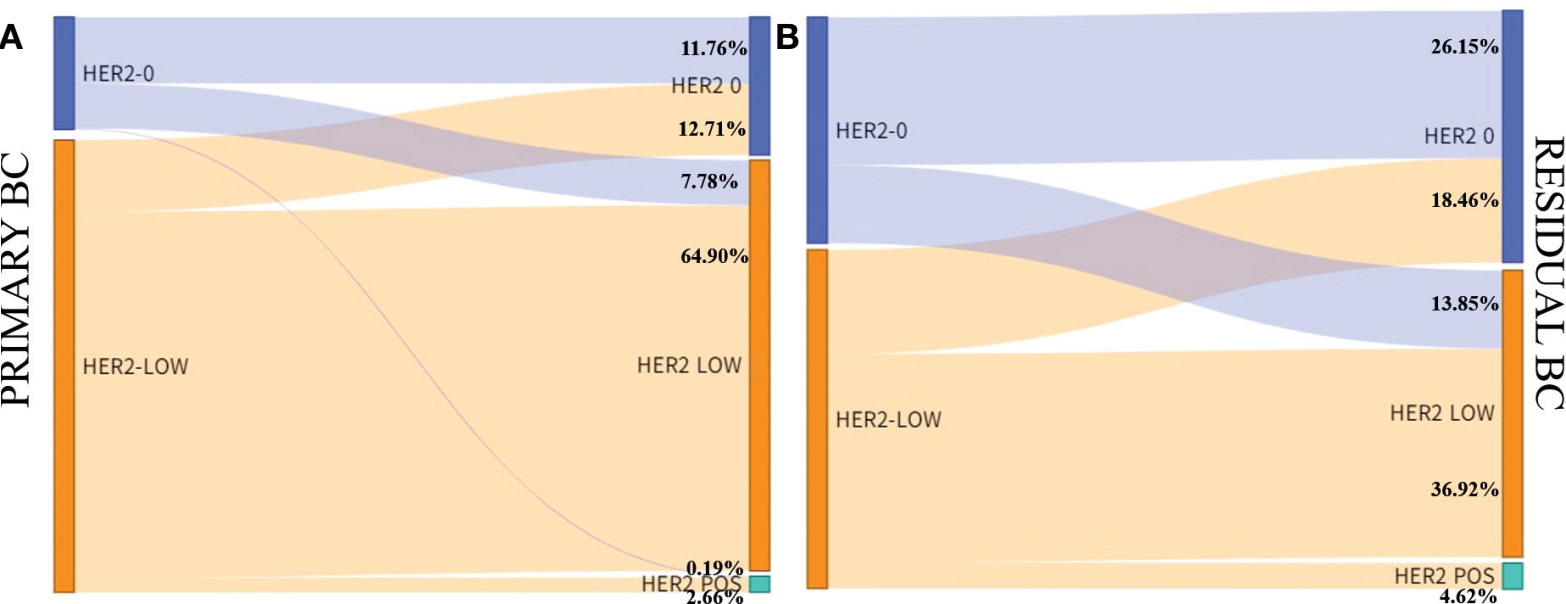
HER2 expression evolution from primary breast cancer to residual according to tumor phenotype in the HER2-low cohort. **(A)** Hormone receptor positive; **(B)** Hormone receptor negative.

In the cohort, there were 592 HER2-negative cases in primary breast cancer, including 527 HR-positive cases and 65 triple-negative cases. Compared with residual breast cancer, the inconsistency rate of HR-positive cases was 123/527, 23.34%; the inconsistency rate of triple-negative cases was 24/65, 36.92% (**Table 4**). The HER2 discordance rate of HR-positive cases was lower than that of triple-negative cases (23.34% vs. 36.92%) (**Figure 5**).

**Table 4:** HER2 expression evolution from primary breast cancer to residual according to tumor phenotype in the HER2-low cohort.

Primary cancer	HER2 expression on residual breast cancer n (%)			
HR-Pos	HER2-zero	HER2-low	HER2-pos	Total
HER2-zero	62 (11.76)	41 (7.78)	1 (0.19)	104 (19.73)
HER2-low	67 (12.71)	342 (64.90)	14 (2.66)	423 (80.27)
Total	129 (24.48)	383 (72.68)	15 (2.85)	527 (100)
HR-Neg				
HER2-zero	17 (26.15)	9 (13.85)	0 (0)	26 (40.00)
HER2-low	12 (18.46)	24 (36.92)	3 (4.62)	39 (60.00)
Total	29 (44.62)	33 (50.77)	3 (4.62)	65 (100)

### HER2 low expression status and AR status

Of the 775 patients in the non-PCR group after neoadjuvant therapy, 677 cases had definite AR status, 432 cases were AR positive, and the positive rate was 63.81%. After neoadjuvant therapy, 677 of the 775 patients in the non-PCR group had definite AR status, and 432 were AR positive. In AR positive cases, 80 cases were HER2-zero, 261 cases were HER2-low and 91 cases were HER2 positive. Among AR negative cases, 67 cases were HER2-zero, 116 cases were HER2-low and 62 cases were HER2 positive. Among them, 524 were HER2-negative. HER2 low expression in both AR positive and AR negative cases were 76.54% and 63.38%, respectively. Chi-square test showed that AR-positive breast cancer had a higher incidence of HER2-low than AR-negative breast cancer (p < 0.01) (**Table 5**).

**Table 5:** Distribution of breast cancer patients with HER2-negative in different AR states.

	HER2 expression n (%)			
		0	Low	*p*
Residual breast cancer n (%)				
AR-positive		80 (15.27)	261 (49.81)	0.002*
AR-negative		67 (12.78)	116 (22.14)	
Total		147 (28.05)	377 (71.95)	

*, P<0.01.

To further analyze the correlation between HER2 low expression and AR status, In 124 HR negative cases after neoadjuvant therapy, 27 cases were HER2-zero, 46 cases were HER2-low, and 51 cases were HER2-positive. There were 45 AR positive cases and 79 AR negative cases. The positive rate of AR was 43.5% in the HER2-low group and 11.1% in the HER2-zero group. HER2-low showed a higher AR positive rate than HER2-zero, and the difference was statistically significant (p < 0.01).

In the TNBC cohort (n=73), the AR positive rate was 31.5%, and the incidence of HER2-low was higher in AR positive breast cancer than in AR negative (86.96% VS 52.00%).

### Clinicopathological features of low HER2 expression

In the HER2-negative group of the non-pCR cohort, compared with the HER2-zero cases, the cases with low HER2 expression had statistical differences in RCB grade, MP grade and the number of metastatic lymph nodes, and the pathological remission rate was lower; and the number of metastatic lymph nodes was more (**Table 6**).

**Table 6:** Baseline patient characteristics stratified by breast residual HER2 status (HER2 0 vs. HER2-low).

Demographics	Total (n=593)	HER2-zero (n=164)	HER2-low (n=429)	χ2	P Value*
Age					
<70 years	583	160 (97.56%)	423 (98.60%)	0.775	0.379
≥70 years	10	4 (2.44%)	6 (1.40%)		
Menopausal status					
Pre/peri-	401	115 (70.12%)	286 (66.67%)	0.647	0.421
Post-	192	49 (29.88%)	143 (33.33%)		
Histology					
Invasive ductal	524	145 (88.41%)	379 (88.34%)	3.414	0.181
Mucinous adenocarcinoma	7	4 (2.44%)	3 (0.70%)		
Other	62	15 (9.15%)	47 (10.96%)		
Maximum diameter after treatment					
<2	268	77 (46.95%)	191 (44.52%)	0.283	0.595
≥2	325	87 (53.05%)	238 (55.48%)		
Ki-67					
≤20%	414	107 (65.24%)	307 (71.56%)	2.247	0.134
>20%	179	57 (34.76%)	122 (28.44%)		
Miller-Payne (MP)					
1	10	1 (0.61%)	10 (2.33%)	12.277	0.015*
2	71	19 (11.59%)	52 (12.12%)		
3	445	116 (70.73%)	328 (76.46%)		
4	54	20 (12.19%)	34 (7.92%)		
5	13	8 (4.87%)	5 (1.16%)		
Residual cancer burden					
I	63	25 (15.24%)	38 (8.86%)	6.589	0.037^*^
II	172	51 (31.10%)	121 (28.20%)		
III	358	88 (53.66%)	270 (62.94%)		
Number of metastatic sites					
<3	316	92 (56.09%)	194 (45.22%)	5.621	0.018^*^
≥3	277	72 (43.91%)	235 (54.78%)		

*, P<0.05.

Among HER2-negative cases, the clinicopathological characteristics of consistent cases (including: HER2-zero and HER2-low) and differential cases (including: HER2-zero to HER2-low and HER2-low to HER2-zero cases) were analyzed (**Table 7**). There were differences in histological type, Ki-67, RCB grade, and the number of lymph node metastasis among the four groups, and the difference was statistically significant (p<0.05).

**Table 7:** Baseline patient characteristics stratified by HER2 status evolution (Differential vs. Consistent).

Demographics	Total (n=574)	Differential (n=129)		Consistent (n=445)		χ2	P Value*
		HER2 0-low	HER2 low-0	HER2 0	HER2 low		
		N=50	N=79	N=79	N=366		
Age							
<70 years	548	49 (98%)	78 (98.73%)	76 (96.20%)	361 (98.63%)	2.376	0.498
≥70 years	26	1 (2%)	1 (1.27%)	3 (3.80%)	5 (1.37%)		
Menopausal status							
Pre/peri-	393	32 (64%)	51 (64.56%)	59 (74.68%)	251 (68.58%)	2.438	0.487
Post-	181	18 (36%)	28 (35.44%)	20 (25.32%)	115 (31.42%)		
Histology							
Invasive ductal	505	46 (92%)	74 (93.67%)	65 (82.28%)	320 (87.43%)	14.693	0.023^*^
Mucinous adenocarcinoma	7	0 (0%)	0 (0%)	4 (5.06%)	3 (0.82%)		
Other	62	4 (8%)	5 (6.33%)	10 (12.66%)	43 (11.75%)		
Maximum diameter after treatment							
<2	265	18 (36%)	35 (44.30%)	40 (50.63%)	172 (46.99%)	2.925	0.403
≥2	309	32 (64%)	44 (55.70%)	39 (49.37%)	194 (53.01%)		
Ki-67							
≤20%	401	30 (60%)	45 (56.96%)	56 (70.89%)	270 (73.77%)	11.248	0.010^*^
>20%	173	20 (40%)	34 (43.04%)	23 (29.11%)	96 (26.23%)		
Miller-Payne (MP)							
1	9	1 (2%)	0 (0%)	0 (0%)	8 (2.19%)	23.137	0.027^*^
2	71	3 (6%)	6 (7.59%)	12 (15.19%)	54 (14.75%)		
3	425	41 (82%)	59 (74.68%)	52 (65.82%)	273 (74.59%)		
4	53	5 (10%)	10 (12.67%)	11 (13.93%)	27 (7.38%)		
5	12	0 (0%)	4 (5.06%)	4 (5.06%)	4 (1.09%)		
Residual cancer burden							
I	59	9 (18%)	10 (12.66%)	11 (13.92%)	29 (7.92%)	13.684	0.033^*^
II	159	10 (20%)	21 (26.58%)	30 (37.97%)	98 (26.78%)		
III	356	31 (62%)	48 (60.76%)	38 (48.11%)	239 (65.30%)		
Number of metastatic sites							
<3	276	21 (42%)	56 (70.89%)	35 (44.30%)	164 (44.81%)	19.221	0.000^*^
≥3	298	29 (58%)	23 (29.11%)	44 (55.70%)	202 (55.19%)		

*, P<0.05.

## Discussion

Neoadjuvant therapy combined with anti- HER2 therapy is an effective treatment option for HER2-positive breast cancer (based on IHC defined as HER2-amplified IHC 3+ or IHC 2+ and ISH amplification). The heterogeneity of HER2 expression before and after neoadjuvant therapy for breast cancer is an area of interest for clinicians and pathologists. HER2-low breast cancer is emerging as a new entity, leading to biological and clinical complexity. Currently, the evolution of HER2-low expression from primary breast cancer to residual breast cancer after neoadjuvant therapy was assessed in a cohort by including the HER2-low category in the characterization of primary and post-neoadjuvant residual tumours.

In a cohort of 775 patients with pathological non-pCR breast cancer after neoadjuvant therapy, HER2-low expressing breast cancers accounted for almost more than half (59.61%) of the entire HER2-negative cohort, which is consistent with available research data (8). Furthermore, in this cohort, the proportion of HER2-low cases in breast cancer samples with residual tumours after neoadjuvant therapy was lower than in breast cancer primaries, and the decrease in HER2-low cases in residual tumours after neoadjuvant therapy compared with breast cancer primaries was mainly due to the fact that HER2-low cases switched to HER2-zero with treatment.

The study showed an association between HR status and HER2-low. HER2-low expression consisted of 80.27% and 60.00% in the HR-positive/HER2-negative cohort and triple-negative cohort for primary breast cancer, respectively, and 74.14% and 53.23% in the HR-positive/HER2-negative cohort and triple-negative cohort for residual breast cancer after NAT, respectively. HER2-low cases were more common in the HR-positive/HER2-negative breast cancer cohort, while HER2-zero cases were more common in the TNBC cohort. This result is consistent with those in previous studies (9, 10) and Schettini et al (9) reported a higher incidence of HR-positive/HER2-negative phenotype than triple-negative phenotype in HER2-low breast cancer. ER levels were higher in the HR-positive/HER2-negative subgroup than in the HER2-low cohort. In conclusion, HR status is a key determinant of the underlying biology of HER2-low breast cancer. The complexity between HER2 and HR pathways may play a key role in biologically defining the HER2-low phenotype (11, 12) However, whether HER2-low can be considered as a separate subtype needs to be further validated in future studies.

Our main objective was to study the evolution of HER2-low from primary breast cancer to residual breast cancer after neoadjuvant therapy. In the whole cohort, the HER2 noncompliance rate was 21.41%, mainly due to the switch from HER2-low to HER2-zero cases. In particular, approximately 17% of patients with HER2-low primary breast cancer exhibited conversion to HER2-zero after neoadjuvant therapy, whereas about 38% of patients with HER2-zero in the primary tumour switched to HER2-low, further confirming the instability of HER2-low expression. The great instability of HER2-low breast cancer was shown in the conversion from HER2-zero phenotype to HER2-low phenotype or from HER2-low phenotype to HER2-zero phenotype and with the use of ADC analogues (13). Therefore, re-testing for HER2 should be recommended for patients with breast cancer after undergoing neoadjuvant therapy. In addition, inconsistent HER2 low expression is primarily driven by the TNBC subgroup, which shows a higher conversion rate compared to the HR-positive/HER2-negative subgroup, especially when considering the conversion of TNBC to the HER2-low phenotype. It should be considered that these patients have exhausted their primary treatment options, including hormonal strategies and chemotherapy after neoadjuvant therapy, but may still benefit from additional therapy. In such cases, those who exhibit low HER2 expression may be ideal candidates for inclusion in ongoing clinical trials of anti-HER2 ADCs. In contrast, although HER2-low expression was observed less frequently in triple-negative cohorts than in HR-positive cohorts, approximately 50% of TNBC patients exhibited an HER2-low status. This result opens up new treatment decisions and opportunities for patients with TNBC.

In general, our findings emphasise the importance of re-testing for HER2 in breast cancer patients after neoadjuvant therapy. Indeed, low HER2 expression can be detected in breast cancer patients with primary HER2-zero after neoadjuvant therapy, thus expanding the treatment options for patients. However, it is unclear whether patients with HER2-low breast cancer who exhibit complete deletion of HER2 expression during disease evolution can still benefit from these new treatment strategies.

In addition, we analysed the pathological remission rates after neoadjuvant therapy in HER2-zero versus HER2-low cases to detect the difference between these two types. The main finding of our study was that HER2-zero and HER2-low expressing tumours are different biological subtypes with distinct clinicopathological features, including differences in HR-positive tumours and in pathological remission rates. Compared to HER2-zero cases, HER2-low cases had statistically different RCB grading, MP grading, and number of metastatic lymph nodes; the pathological remission rates were lower, and the number of metastatic lymph nodes was higher. We also analysed the clinicopathological characteristics of concordant cases (including HER2-zero and HER2-low cases) versus discrepant cases (including HER2-zero to HER2-low, and HER2-low to HER2-zero cases) in the HER2-negative cohort. There were differences in histological staging, Ki-67 index, MP grading, RCB grading, and number of lymph node metastases among the four groups of cases; the differences were statistically significant (p < 0.05).

The biological staging of breast cancer has always been based on HR status (HER2-negative and HER2-positive) (14). Our study not only confirmed the correlation between HER2 low and HR status, but also closely correlated with AR status. In addition, in order to confirm the correlation between low HER2 expression and AR positivity, we excluded the influence of HR status and conducted the study on the TNBC cohort. The incidence of HER2 low expression in AR positive cohort was significantly higher than that in AR negative cohort. This has not been shown in other studies.In breast cancer, these new subtypes can be distinguished by the standardized pathological assessment of HRs and HER2, especially in HER2-low breast cancer. This will lead to more complex breast cancer subtypes and provide new targeted therapeutic options to improve breast cancer prognosis.

This study also has certain limitations, because the collected cases were recent breast cancer patients, whose prognostic information was not obtained. Therefore, some biological characteristics of HER2-low breast cancer were not studied.

## Conclusion

HER2-low breast cancer is highly unstable during disease evolution and has certain biological characteristics, and breast cancer with HER2-low positivity has certain biological characteristics, which are correlated with positive HR and positive AR. Whether HER2-low breast cancer can be regarded as a new subtype still needs to be confirmed by more studies. At the same time, re-detection of HER2 in breast cancer after neoadjuvant therapy may bring new treatment opportunities for a certain proportion of patients.

## Data availability statement

The raw data supporting the conclusions of this article will be made available by the authors, without undue reservation.

## Ethics statement

The studies involving human participants were reviewed and approved by the Ethics Committee of the Fourth Hospital of Hebei Medical University: 2022KS023. Written informed consent for participation was not required for this study in accordance with the national legislation and the institutional requirements.

## Author contributions

Conception and design: YL. Administrative support: YL. Provision of study materials or patients: JS. Collection and assembly of data: JS. Data analysis and interpretation: JS. Manuscript writing: All authors. Final approval of manuscript: All authors. All authors contributed to the article and approved the submitted version.
